# Mammalian interspecies substitution of immune modulatory alleles by genome editing

**DOI:** 10.1038/srep21645

**Published:** 2016-02-22

**Authors:** Simon G. Lillico, Chris Proudfoot, Tim J. King, Wenfang Tan, Lei Zhang, Rachel Mardjuki, David E. Paschon, Edward J. Rebar, Fyodor D. Urnov, Alan J. Mileham, David G. McLaren, C. Bruce A. Whitelaw

**Affiliations:** 1 The Roslin Institute and R(D)SVS, Easter Bush Campus, University of Edinburgh, Edinburgh, EH25 9RG, UK; 2 Sangamo BioSciences, Point Richmond Tech Center, 501 Canal Boulevard, Suite A100, Richmond, CA 94804, USA; 3 Genus plc, 1525 River Rd., DeForest, WI 53532, USA

## Abstract

We describe a fundamentally novel feat of animal genetic engineering: the precise and efficient substitution of an agronomic haplotype into a domesticated species. Zinc finger nuclease in-embryo editing of the RELA locus generated live born domestic pigs with the warthog RELA orthologue, associated with resilience to African Swine Fever. The ability to efficiently achieve interspecies allele introgression in one generation opens unprecedented opportunities for agriculture and basic research.

Classical animal breeding utilises sequence variation across the entire genome. Offspring resulting from mating of two animals have a genotype which is a mix of both parents plus *de novo* mutations. In agriculture beneficial genotypes and their encoded traits are captured conferring genetic improvement. This process is time consuming, requires multiple crosses, and relies on the presence of the desired genetic variation in the breeding population; variation that is eliminated during the breeding process cannot be exploited.

The development of targeted genome editing^1^, pioneered by the zinc finger nuclease (ZFN) approach^2^, now enables variation not present within a given population to be used. This approach relies on engineered nucleases to direct a double-strand break (DSB) to a specific target sequence. When used in combination with an investigator-provided DNA template, specified changes can be introduced into the chromosome in a homology-based process during repair of the DSB. In this way the original target sequence can be exchanged for a new sequence^3^, enabling single allele introgression into the target animal population in one generation^4^.

While single point mutations have been found to confer key phenotypic differences during domestication of wild species^5^, in many cases, multiple point mutations within the same locus are thought to be responsible. A representative, agronomically important example is variation in *RELA*^6^. The domestic pig is highly susceptible to infection by African Swine Fever Virus, in contrast to present-day pig species found in Africa. We have earlier identified three amino acid differences between warthog (*Phacochoerus africanus*) and domestic pig (*Sus scrofa*) RELA as potentially responsible for the distinct response of the two species to this virus^6^.

Pioneering work by the Jasin laboratory demonstrated that a single DSB in mammalian cells can lead to the transfer of a panel of single nucleotide polymorphisms (SNPs) forming an uninterrupted, short (<200 bp) haplotype to the chromosome from an extrachromosomal repair template^7^. Because the warthog haplotype bearing the three SNPs spans a relatively short [251 base pairs] distance, we reasoned that we could use genome editing to introduce this entire haplotype via a single nuclease-induced DSB. To the best of our knowledge, such editing-driven haplotype introgression had not been previously reported in live born animals of any mammalian species. We now demonstrate that this can be achieved efficiently in domestic pig by direct injection into the zygote cytoplasm. Strikingly, we observe single-step biallelic haplotype transfer by genotyping of piglets.

## Results

**Figure 1:**
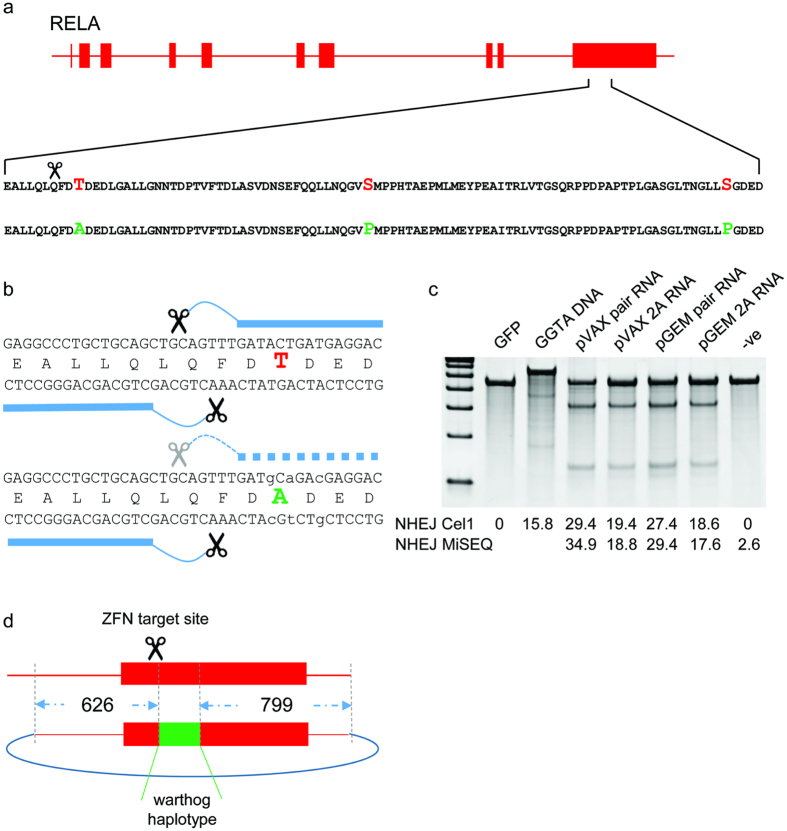
Design strategy for creation of a DSB and subsequent HDR of the domestic pig *RELA*. (**a**) Depiction of the RelA gene with exons as solid bars. An expanded view of a region of the final exon highlights the 3 amino acid differences between domestic pig (red) and warthog (green). Scissors indicate the intended site of the DSB. (**b**) DNA sequence, with solid bars representing ZFN binding sites on the domestic pig sequence. Lower panel indicates sequence changes that would concurrently change the domestic pig threonine to the warthog alanine whilst at the same time prevent recutting by the ZFN. (**c**) *in vitro* comparison of ZFN delivery strategies and cutting efficiencies in porcine PK15 cells. Control cells were transfected with either a plasmid encoding GFP or a previously published plasmid encoding a ZFN pair targeting GGTA (first 2 lanes) or remained untransfected (final lane). Experimental transfections were with a variety of *in vitro* transcribed mRNAs encoding a ZFN pair targeting RELA. Comparisons were between post transcriptional polyadenylation (pVAX) or an inbuilt polyadenylation track (pGEM), and between individual mRNAs delivered in pairs compared to a single mRNA encoding both ZFNs interspersed by a 2A site. Cutting efficiencies were calculated using Cel1 nuclease assay and MiSEQ sequencing of the target locus. (**d**) Design of HDR template, with 626 bp and 799 bp homology arms flanking a 251bp region, including 5 base changes to convert the domestic pig sequence to the warthog haplotype.

ZFNs can be engineered to induce a DSB at any genomic position. In this specific case, nuclease design considerations were informed by the need to transfer an entire 251 bp haplotype bearing multiple SNPs. This suggested a strategy (Fig. 1a) in which a ZFN is engineered for a region immediately upstream of the haplotype-marked stretch, in the hope that single-sided invasion of the repair template by the upstream chromosome arm would then lead to a synthesis-dependent strand annealing-based transfer of the entire downstream haplotype to the endogenous locus^7^.

We engineered a ZFN heterodimer that binds to the region flanking 1330 to 1338 bp relative to the translational start site in the porcine *RELA* cDNA sequence (NM_001114281). We compared two formats of an expression construct for the ZFNs: two plasmids, each encoding a single ZFN monomer, and one plasmid that encodes both ZFN monomers spanned by a ribosome stuttering signal or a 2A peptide^8^,^9^. We transfected these plasmids into a transformed cell line (PK15) established from the domestic pig, and compared genome editing efficiencies via the Surveyor/Cel-1 endonuclease assay^10^. We observed comparable on-target editing driven by either expression construct configuration (Fig. 1c). The editing efficiency driven by the *RELA*-directed ZFNs nearly doubled what was seen with ZFNs successfully used to obtain live pigs bearing a disruption of the GGTA gene^11^, suggesting that these nucleases may be well-suited for in-embryo editing.

A robust combination of efficient on-target marking and minimal toxicity to early embryogenesis requires the delivery of nuclease-encoding mRNA to the embryo^12^. We transferred the ORFs encoding the *RELA* ZFNs into two distinct vectors for *in vitro* mRNA production (pVAX, which requires *in vitro* polyadenylation, and pGEM, which contains a polyA track of defined length). For both vectors, we generated constructs bearing single ZFNs, and constructs bearing both ZFNs on the same ORF separated by a 2A signal. Capped and polyadenylated mRNA was then *in vitro* transcribed from all constructs, and the on-target editing efficiency assessed by transient transfection into pig PK15 cells. This was followed by Surveyor/Cel-1 and deep-sequencing based assays to measure the percentage of edited chromatids. As shown in Fig. 1c, robust editing efficiency, in all cases exceeding that driven by positive control ZFNs, was obtained with all four vector/ORF configurations. We have previously shown that delivery of engineered nucleases to the cytoplasm of livestock zygotes can result in the production of small insertions or deletions (indels) due to non-homologous end-joining-driven (NHEJ) break repair at the target site^13^,^14^,^15^. In order to investigate whether this delivery method could also result in HDR if combined with a DNA template, we co-injected porcine zygotes with mRNA encoding the pair of ZFN and a single stranded oligodeoxynucleotide (ssODN^16^) or plasmid DNA bearing the warthog SNPs. Injected zygotes were transferred to recipient gilts^14^.

To determine whether the nucleases drove targeted editing of pig *RELA*, ear notches were taken from piglets 2 days postpartum and genomic DNA was prepared. PCR spanning the target locus and sequencing of these products was used to identify alleles bearing either small indels (a result of NHEJ) or specific point mutations (a result of HDR events). No indels were observed at the ZFN target site in any of the animals genotyped; this contrasts both with our ability to obtain edited animals bearing NHEJ-generated alleles, and our earlier experience with genome editing in pig^13^,^14^.

**Figure 2:**
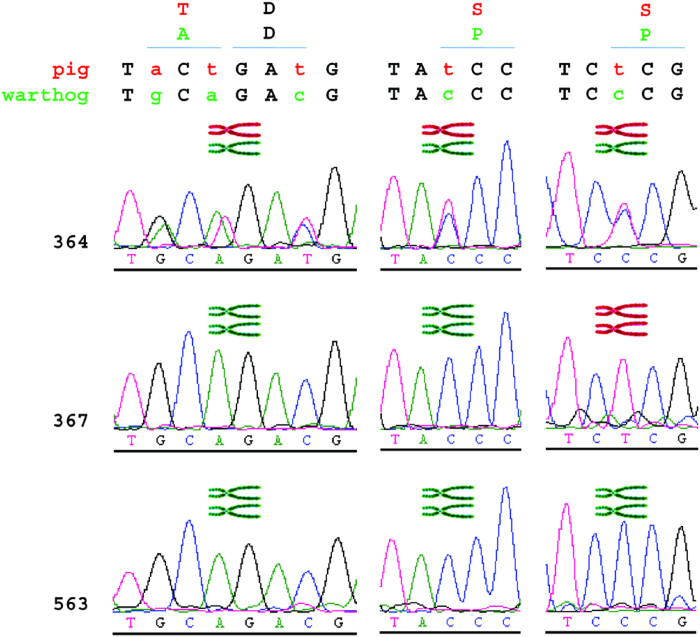
Sequence analysis of live born piglets. The sequence of both the domestic pig and warthog encoding the three observed amino acid differences is shown above, with sequence traces from individual animals below. Inset pictures of chromosomes indicate the allelic makeup at each position in each animal (domestic pig allele-red; warthog allele-green).

The lack of indels at the nuclease target site was not due to a failure of the genome editing process itself, as three live piglets bore HDR-generated alleles of *RELA* (Supplementary Table S1). All three occurred in the cohort of 46 animals injected with ZFN-encoding mRNA and a plasmid repair template. In contrast, no HDR events were observed in the 39 live born pigs where ssODN was provided as the HDR template. Sanger sequencing of PCR products spanning the target locus of the HDR positive pigs showed that piglet 364 was heterozygous at each of the 5 base changes encoded by the plasmid template, thus representing full haplotype introgression (Fig. 2). Piglet 367 was homozygous for 4 base changes proximal to the ZFN target site, encoding the first 2 intended amino acid modifications, but was wild type at the final position. Remarkably, piglet 563 was homozygous for all 5 base changes. No footprint was observed in any of the three animals identifying the intersection between the HDR plasmid construct sequence and the pig genome. Unexpectedly, PCR of piglet gDNA for the beta lactamase gene present in the HDR plasmid revealed 367 and 563 to harbour randomly integrated plasmid in addition to HDR. It is intended to segregate plasmid insertion from HDR through standard breeding for subsequent generations.

## Discussion

Livestock breeding has enabled significant increases in animal productivity since the pioneering work of Robert Blackwell in the late eighteenth century. The challenge ahead is to accelerate this improvement process to meet the demands imposed on agriculture through climate change, resource and land availability in conjunction with the increase in human population. Genome editing technology has the potential to revolutionize livestock breeding^4^, and targeted gene knockout in several livestock species has been attained using multiple distinct designed nuclease platforms, including ZFNs, TAL effector nucleases, and CRISPR/Cas9^11^,^13^,^15^,^17^,^18^,^19^. In the present study we significantly expand the genome editors’ toolbox to include the targeted transfer of an entire haplotype. Specifically, through homology dependent repair of a ZFN-induced break using a plasmid repair template we have introgressed an allele of the *RELA* gene between pig species, producing live piglets both heterozygous and homozygous for the desired haplotype. Thus alleles found in distinct animal populations can now be exchanged, providing a new route for the animal breeder to benefit from previously unavailable genetic variation.

## Methods

### ZFN Design and Validation

ZFNs against the indicated position of the pig RELA gene were designed and assembled using an archive of pre-validated two-finger modules as described^2^. The ORFs were cloned into expression vectors harboring enhanced obligate heterodimer forms of FokI^20^ optimized for delivery in DNA form and for production of *in vitro* transcribed mRNA (Vierstra *et al.*, in press). ZFN target sequences and DNA recognition helices are described in Supplementary Table 2. Pig PK15 cells were electroporated using ZFN-encoding DNA or mRNA as described, genomic DNA harvested 48 hrs following electroporation, and percentage of chromatids bearing indels was measured using Surveyor/Cel1 as described^10^ or deep sequencing on the Illumina platform (Vierstra *et al.*, in press).

### Design and construction of HDR templates

A 96-mer ssODN was designed spanning the target site of the ZFN and containing two base changes encoding the desired T448A conversion (Fig. 1a) plus a third silent base change to prevent ZFN recutting of any introgressed alleles. The plasmid DNA template was designed with the same three base changes as the ssODN at the ZFN target site, with additional single base changes encoding the S485P and S531P (Fig. 1b). This 251 bp central domain containing all five base changes was flanked by homology arms of 626 bp and 799 bp, 5′ to the first base change and 3′ to the final base change respectively.

### Zygote injection and transfers

All animal work was approved under UK Home Office licence after review by the University of Edinburgh’s Animal Ethics Committee and was carried out in accordance with the approved guidelines. Embryos were produced from Large-White gilts that were approximately 9 months of age and weighed at least 120 kg at time of use. Super-ovulation was achieved by feeding, between day 11 and 15 following an observed oestrus, 20 mg altrenogest (Regumate, Hoechst Roussel Vet Ltd) once daily for 4 days and 20 mg altrenogest twice on the 5th day. On the 6th day, 1500 IU of eCG (PMSG, Intervet UK Ltd) was injected at 20:00 hrs. Eighty three hours later 750 IU hCG (Chorulon, Intervet UK Ltd) was injected. Donor gilts were inseminated twice 6 hours apart after exhibiting heat generated following super-ovulation. Embryos were surgically recovered from mated donors by mid-line laparotomy under general anesthesia on day 1 following oestrus into NCSU-23 HEPES base medium. Embryos were subjected to a single 2–5pl cytoplasmic injection of the pVAX single mRNAs at 2 ng/ul or 4 ng/μl with ssODN or plasmid template respectively. Recipient females were treated identically to donor gilts but remained unmated. Following ZFN injection, fertilized embryos were transferred to recipient gilts following a mid-line laparotomy under general anaesthesia. During surgery, the reproductive tract was exposed and embryos were transferred into the oviduct of recipients using a 3.5 French gauge tomcat catheter. Litter sizes ranged from 1–13 piglets.

### Genotyping

Genomic DNA was prepared from ear biopsy taken from piglets 2 days postpartum. PCR amplification with AccuPrime HiFi was conducted with primers oSL1 (gggtacaaagaggggtgagg) and oSL152 (agctaggggtttccactggt) which bind out-with the 5′ homology arm and the 3′ homology arm of the plasmid respectively. Cycling was 95 °C for 120 seconds then 40 cycles of 94 °C for 30 seconds, 63 °C for 30 seconds and 68 °C for 120 seconds, followed by primer extension of 68 °C for 5 minutes. Purified PCR products were directly sequenced.

## Additional Information

**How to cite this article**: Lillico, S. G. *et al.* Mammalian interspecies substitution of immune modulatory alleles by genome editing. *Sci. Rep.* **6**, 21645; doi: 10.1038/srep21645 (2016).

## Supplementary Material

Supplementary Information
