# Supplementary material for: Mammalian interspecies substitution of immune modulatory alleles by genome editing
Source: Sci Rep. 2016 Feb 22;6:21645. doi: 10.1038/srep21645 (PMC4761920; doi:10.1038/srep21645)
Supplement: Supplementary Information [file srep21645-s1.pdf]

Mammalian interspecies substitution of immune modulatory alleles by genome editing

Simon G. Lillico[1], Chris Proudfoot[1], Tim J. King[1], Wenfang Tan[1], Lei Zhang[2], Rachel Mardjuki[2], David E. Paschon[2], Edward J. Rebar[2], Fyodor D. Urnov[2], Alan J. Mileham[3], David G. McLaren[3] and C. Bruce A. Whitelaw*[1]

Supplementary Table S1. Summary of pig zygote injections.

Construct	N° zygotes injected	N° recipients	N° live piglets	NHEJ	HDR
ZFN + ssODN	95	4	39	0	0
ZFN + plasmid	272	6	46	0	3

Supplementary Table S2. ZFN target sites at pig RELA locus and designed zinc finger helices sequences.

ZFN Binding Sequence (underlined)	ZFN ID	Finger 1	Finger 2	Finger 3	Finger 4	Finger 5
AGAGGCCCTGCTGCAGCTGCAGTTTGATACTGATGAGGACC	48307	DRSDLSR	RSDNLTR	TSGNLTR	LRQDLNK	TSSNLSR
TCTCCGGGACGACGTCGACGTCAAACTATGACTACTCCTGG	48304	AMQTLRV	DRSHLAR	RSDNLSE	KRCNLRC	RSAVLSE
